# Relation of blood lead levels and lead in gasoline: an updated systematic review

**DOI:** 10.1186/s12940-022-00936-x

**Published:** 2022-12-27

**Authors:** Ruth C. Angrand, Geoffrey Collins, Philip J. Landrigan, Valerie M. Thomas

**Affiliations:** 1 Department of Internal Medicine, Stony Brook University Hospital, Stony Brook, NY USA; 2 Department of Population Health Science and Policy, Mount Sinai School of Medicine, New York, NY USA; 3 Biology Department and Global Observatory on Planetary Health, Boston College, Boston, MA USA; 4 Centre Scientifique de Monaco, Monaco, MC Monaco; 5 H. Milton Stewart School of Industrial and Systems Engineering, and School of Public Policy, Georgia Institute of Technology, Atlanta, GA USA

**Keywords:** Petrol, Time trend, Unleaded, Africa, Asia, Europe, North America, Oceania, South America

## Abstract

**Background:**

Millions of tons of lead were added to gasoline worldwide beginning in 1922, and leaded gasoline has been a major source of population lead exposure. In 1960s, lead began to be removed from automotive gasoline. Removal was completed in 2021.

**Objectives:**

To determine whether removal of lead from automotive gasoline is associated with declines in population mean blood lead levels (BPb).

**Methods:**

We examined published studies that reported population blood leaded levels for two or more years, and we calculated average concentrations of lead in gasoline corresponding to the years and locations of the blood lead level measurements.

**Results:**

Removal of lead from gasoline is associated with declines in BPb in all countries examined. In some countries, BPb continues to fall after lead has been eliminated from gasoline. Following elimination of lead from gasoline, BPb less than 1 μg/dL have been observed in several European and North American countries, and BPb less than 3 μg/dL have been documented in several studies from South America.

**Discussion:**

There remain many countries for which no multi-year studies of populations BPb have been identified, including all of Central America, high population countries including Pakistan and Indonesia, and major lead producers including Australia and Russia.

**Conclusion:**

Removal of lead from gasoline has been a public health success. Elimination of lead from gasoline has enabled many countries to achieve population mean BPb levels of 1 μg/dL or lower. These actions have saved lives, increased children’s intelligence and created great economic benefit in countries worldwide.

**Supplementary Information:**

The online version contains supplementary material available at 10.1186/s12940-022-00936-x.

## Key messages

1. The addition of lead to gasoline was a catastrophe for global health. It caused neurodevelopmental disability with diminished intelligence and disordered behavior in millions of children, as well as neurobehavioral deficits and premature deaths from cardiovascular and kidney disease in millions of adults, and great economic losses.
2. WHO and UNEP deserve enormous credit for having successfully completed the removal of lead from automotive gasoline in all countries around the world. This work was accomplished under the leadership of the Partnership for Clean Fuels and Vehicles (PCFV) led by the United Nations Environment Programme and the World Health Organization [1].
3. It is important to prevent the addition of other harmful substitutes to gasoline such as benzene or manganese in lieu of lead [2].

The lead pandemic provides an object lesson of the dangers of ignoring early warnings on potential danger [3] and of permitting wide use and dissemination of chemicals that have not been adequately tested for potential toxicity [4]. The use of lead in gasoline is a classic example of our society’s willingness to adopt a promising but unproven new technology without heed to its possible consequences. We made the same error with chlorofluorocarbon (CFCs), and we are at risk of making it again if we adopt fuel additives containing manganese, a known neurotoxicant [2]. For our children’s future [5], we must do better.

## Introduction

In the past century, millions of tons of lead were added to gasoline worldwide [6]. The result has been a global pandemic of lead poisoning with damage to health, impairment of cognitive function, reduction in life expectancy, and premature death of millions of persons. This pandemic began in 1922 when lead in the form of tetraethyl lead was first added to motor fuels, accelerated after World War II, and peaked in the 1970s and 1980s. Neurodevelopmental impairment with IQ loss, shortened attention span, dyslexia, attention deficit/hyperactivity disorder, school failure, and increased future risk for drug abuse, criminal behavior and incarceration are the main health consequences in children [7-11]. Neurobehavioral impairment, hypertension, renal disease, cardiovascular disease, stroke and premature death are the health consequences in adults [12, 13]. It is now known that no level of lead is safe [14, 15].

The dangers of adding lead to gasoline first became evident in the 1920’s when a cluster of cases of acute neuropsychiatric disease appeared among workers occupationally exposed to tetraethyl lead at a refinery in Bayway, New Jersey, USA; 80% of the affected workers developed convulsions and five died [16]. These warnings were, however, ignored and after a brief pause in production, the addition of lead to gasoline resumed [3, 16]. At peak use in the 1970s and 1980s, virtually all countries around the world added lead to motor fuels [17]. Levels of lead were found to be elevated in cities and along roadways [18]. Geochemical studies conducted in the high Arctic documented an unprecedented increase in atmospheric deposition of lead into the Greenland ice cap [19]. These findings have been confirmed more recently by studies of lead deposition in Alpine glaciers [20].

Lead began to be removed from gasoline in the late 1960s, first in the Soviet Union, then in Japan and the United States [21]. The US Environmental Protection Agency mandated removal of lead from gasoline beginning in 1975 because of two discoveries – first, the recognition that lead released to the environment by the combustion of leaded gasoline was causing widespread lead poisoning in children [7, 8], and secondly, the realization that lead in gasoline was destroying the catalytic converters required on all new cars in compliance with the Clean Air Act Amendments of 1970 [22].

In the United States, the removal of lead from gasoline was followed by a more than 90% reduction in mean blood lead concentration between 1976 and 1995 [23]. This decline closely paralleled year-to year reductions in the amount of lead added to gasoline. The percentage of children in the United States aged 1–5 years with blood lead levels greater than or equal to 10 μg/dl fell from nearly 80% in the late 1970s to less than 5% in the early 1990s [24]. Other factors that may have contributed to the decline in blood lead levels in the United States were increased professional and public awareness of the dangers of low-level lead exposure and the banning of lead from domestic paint and plumbing supplies; however there is a close temporal parallel between the reduction of lead in gasoline and the decline in blood lead levels. The mean blood lead level in the United States today is less than 1 μg/dL [25].

Our data indicate that all countries, in all regions of the world have now eliminated lead from gasoline used in motor vehicles. Algeria, the last country to remove lead from automotive gasoline, did so in 2021. Declines in blood lead levels following removal of lead from gasoline have been reported in numerous countries and cities [26]. Lead is still used in racing cars and in aviation gasoline for small, piston-engine aircraft [27].

To assess the impact on global health of reducing lead levels in gasoline, Thomas et al. analyzed 17 studies from 5 continents and reported their findings in 1999 [28]. They compared reductions in blood lead levels in cities and countries with reductions in the mean concentration of lead in gasoline and found a strong linear correlation between the two with a median correlation coefficient of 0.94. Data on air lead concentrations were also available in some of the same locations, and in those places, correlations were observed between declining concentrations of lead in gasoline and reductions in air lead levels. In most locations that completely removed lead from gasoline, mean blood levels fell to approximately 3 μg/dL. Higher residual blood lead levels were observed in locations with widespread exposure to lead from sources other than leaded gasoline such as exposures to lead mining and smelting, lead battery recovery operations, lead-contaminated waste sites, e-waste recovery operations, lead-glazed pottery, and lead-containing cosmetics and medications [29–34].

Now to extend, update and corroborate the 1999 analysis by Thomas et al. [28], we have gathered 38 additional reports. For those reports as well as for earlier publications, we have systematically examined the relationships between reductions in levels of lead in gasoline and declines in population blood lead levels. This work was undertaken as a component of an effort led by the World Health Organization to develop guidelines on the prevention and management of lead exposure.

## Methods

### Search strategy for blood lead studies

The populations of interest are the general populations, children and adults, not known to be living near industrial lead sites or occupationally exposed, for which there are serial measurements of blood lead levels so that changes in blood lead levels can be observed over time as levels of lead in gasoline change. Comparison is provided both with time periods in which use of lead in gasoline lead did not change, as well as with time periods with quantitatively different changes in gasoline lead. Relevant outcomes include the amounts and rates of change in blood lead levels. In consultation with an experienced research librarian, we developed search strategies using the key words “lead blood level” and “gasoline.” We searched for full-text articles in the following databases: PubMed, Global Health in Ovid SP, EMBASE, and the Cochrane Library.

We developed and refined a data extraction sheet. One author extracted data from each study included in the analysis, and the second author checked the extracted data to ensure accuracy. Disagreements were resolved by discussion between the two review authors; if no agreement could be reached, a third author was available to adjudicate. However, all disagreements were resolved without the need for a third review. If an article identified through the databases could not be found, the corresponding author listed in the research article was contacted for the full-text article. If there was confusion about the contents of a research article, the corresponding author was contacted for further clarification. If the corresponding author was unable to be contacted, the principal investigator for the respective research article, subsequently followed by any additional authors listed, were contacted.

We included observational studies that measured serial blood lead levels (BLLs) in populations over a time span of at least 1 year. We also included studies that compared primary data with historical data. We included studies examining populations of both males and females, all age groups, and both urban and rural communities. Finally, studies in this systemic review had to include BLLs as their primary outcome.

Through this systematic search, we identified a total of 1696 records through database searching. After removing duplicates, 1264 records were screened and 874 articles were excluded based on the title and/or the abstract. Three hundred and ninety articles were then assessed for eligibility. Three hundred and fifty-one articles were excluded for reasons, including:

- The article was published in a language not accessible to the reviewers.
- The article was not available (only an abstract or poster was available).
- The article was not relevant to the systematic review.
- The article was a review paper and did not provide any original data.
- The article provided no quantitative outcome data.
- The article did not have data for at least two time periods separated by at least 1 year.

**Fig. 1:**
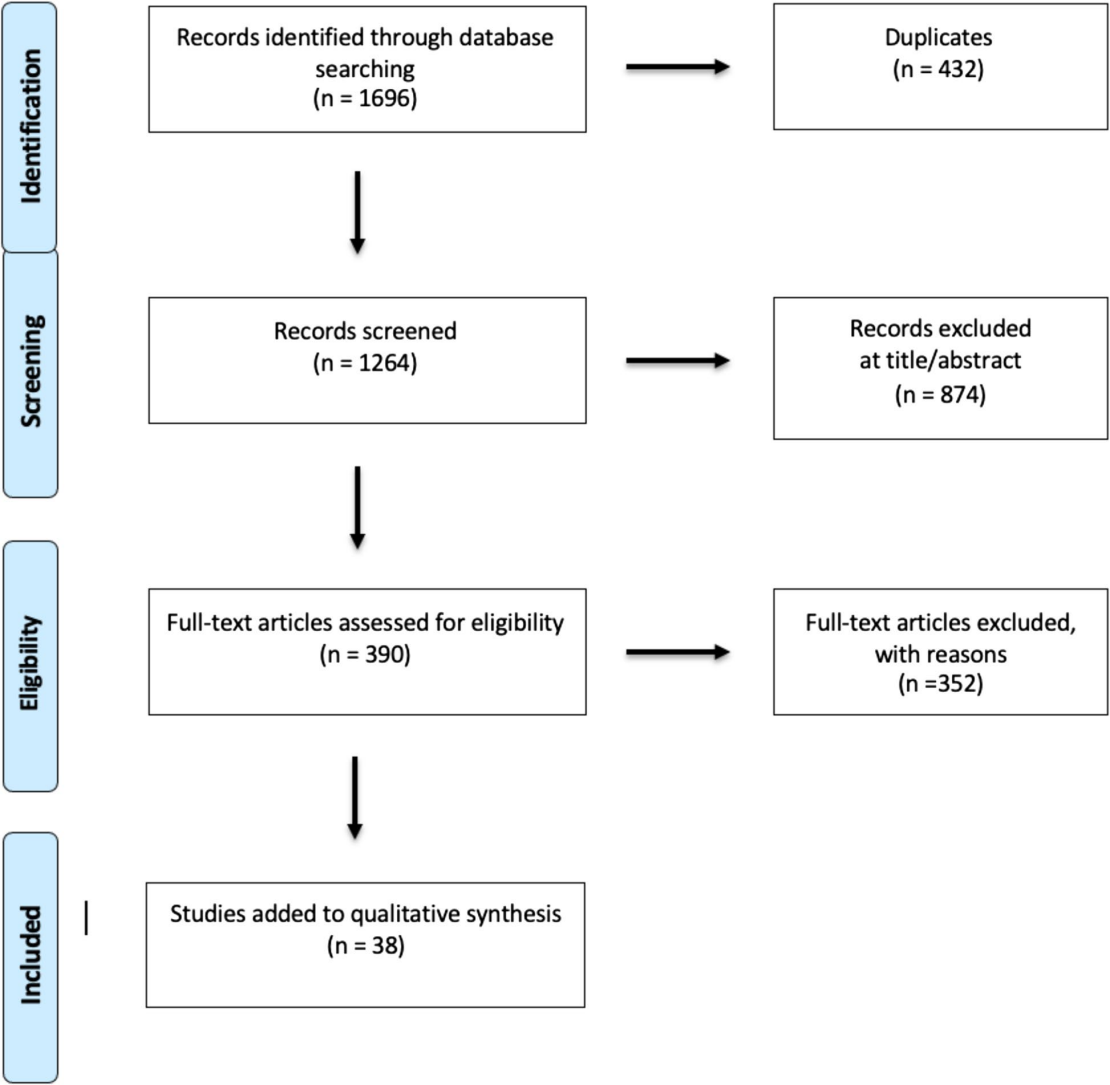
Study selection flow chart

No evaluations of study quality or the possibility of bias other than the above criteria were applied. The criterion of providing blood lead measurements for more than one time period separated by at least 1 year is a significant marker of achievement, requiring ongoing laboratory and analysis capability. In sum, 38 articles were added to the systematic review of changes in blood lead levels related to changes in the lead content of gasoline published previously by Thomas et al. [28]. The data extracted from these articles was population blood lead level for each year reported, population location, and the population age range (Fig. 1).

### Database development for gasoline lead use

Measures of gasoline lead levels were calculated using data from Octel Ltd. [35], the world’s principal manufacturer of tetraethyl lead. Octel reports are available for the years 1968, 1969, 1971, 1975, 1976, 1978, 1979, 1981, 1982, 1983, 1984, 1991, 1992/3, 1994, 1995, and 1996. 1995 is the last year in which Octel included gasoline lead concentrations in its reports. The Octel reports provide data at the national level, and usually include the amount of gasoline and the concentration of lead in both premium and in regular gasoline grades. We use the weighted average of the concentration in premium and regular grades of gasoline. The Octel data were supplemented and cross-checked with data available from other sources, including data reported in the blood lead studies. For years after 1995 we used data developed from extensive literature review.

To characterize the use of lead in gasoline we consider two metrics, the total amount of lead used in gasoline, and the average concentration of lead in gasoline, calculated as the consumption-weighted average lead concentration of all gasoline grades sold, including unleaded gasoline.

Table S1 in the Supplemental Material summarizes the data on blood lead and gasoline lead included in this study.

### Statistical analysis

Each included study reports data on population blood lead concentrations for at least 2 years for the same or similar location and for a similar population group. For each year with a reported population blood lead level, we calculated the average gasoline lead level, including both leaded and unleaded gasoline, on a national level. In cases in which the reported population blood lead levels covered a combined two-year period, in which case we use the average gasoline lead concentration over the two-year period.

Studies are from different countries and locations, and the population cohorts differ by age and location, and thus we do not combine results from different studies, and we do not extrapolate or fit the data. There are measurement errors in both the blood lead values and the gasoline lead values. We estimate the error in the gasoline lead values as 10-20%.

## Results

Results are shown by continent.

**Fig. 2:**
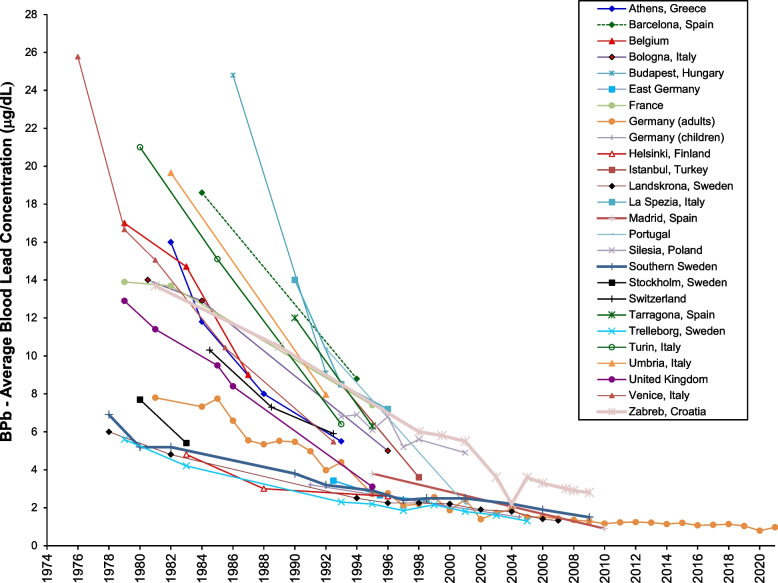
Population blood lead levels by year in Europe. Twenty-eight studies are shown; each marker is one data point; each line connects the data for a single study

**Fig. 3:**
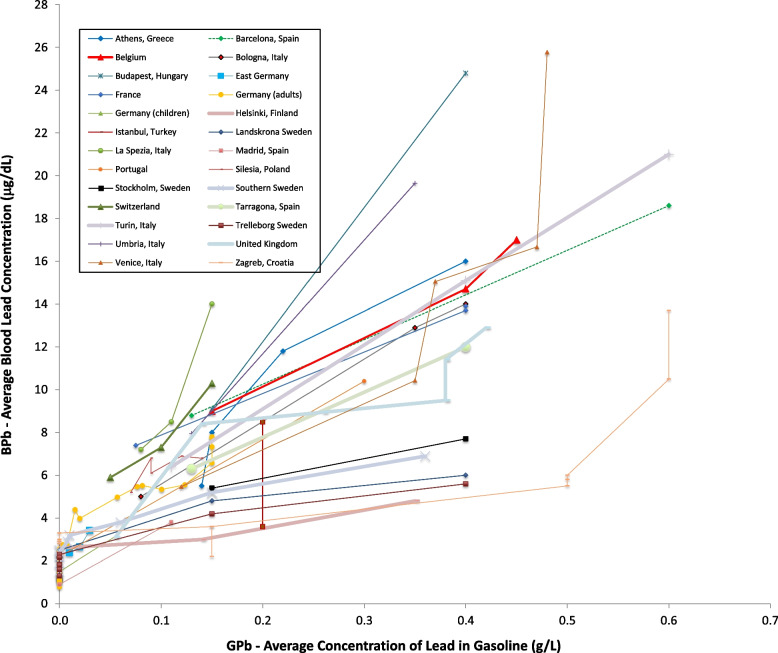
Population blood lead levels versus gasoline lead concentrations in Europe. Twenty-eight studies are shown; each marker is one data point; each line connects the data for a single study

Figures 2 and 3 show results for Europe, for which there have been more studies in different countries of blood level levels over time than for any other continent. Studies from several countries – Germany [36], Sweden [37], and Spain [38]– show blood lead concentrations of less than 2 μg/dL. All of the European studies show decreased blood lead concentrations over time. Figure 3 shows that in the majority of locations, blood lead concentrations fell in close temporaal concordance with gasoline lead concentrations [39–60]. In Istanbul blood lead levels fell without a reduction in gasoline lead; Furman [61] reports that there had been a substantial decrease in gasoline lead levels several years before the reported measurements were made. Also to some extent in Venice and Zagreb, blood lead levels fell without a reduction in gasoline lead [62, 63]. This may indicate reduction in other sources of exposure, as well as uncertainty in the use of lead in gasoline. The data for several countries show continued blood lead reduction after the complete phase out of gasoline lead; this is expected, as lead remains in the body for years [64].

Even though there are numerous European studies, there are countries for which there are no data on how blood lead concentrations have changed over time. There are no studies over time from Russia, Ukraine, Romania, Netherlands, Czech Republic, and others. Russia is a major lead producer, but nonetheless took early action to eliminate lead from gasoline in major cities [65].

**Fig. 4:**
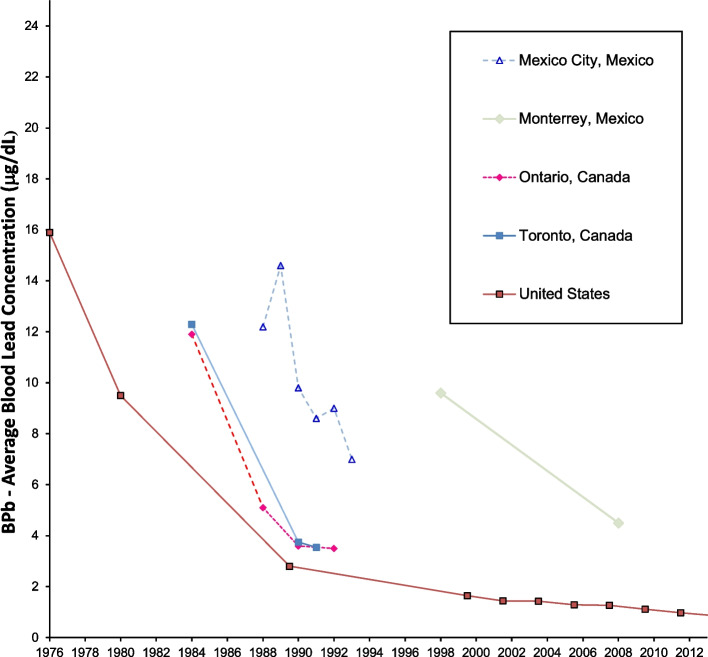
Population blood lead levels by year in North America. Five studies are shown; each marker is one data point; each line connects the data for a single study

**Fig. 5:**
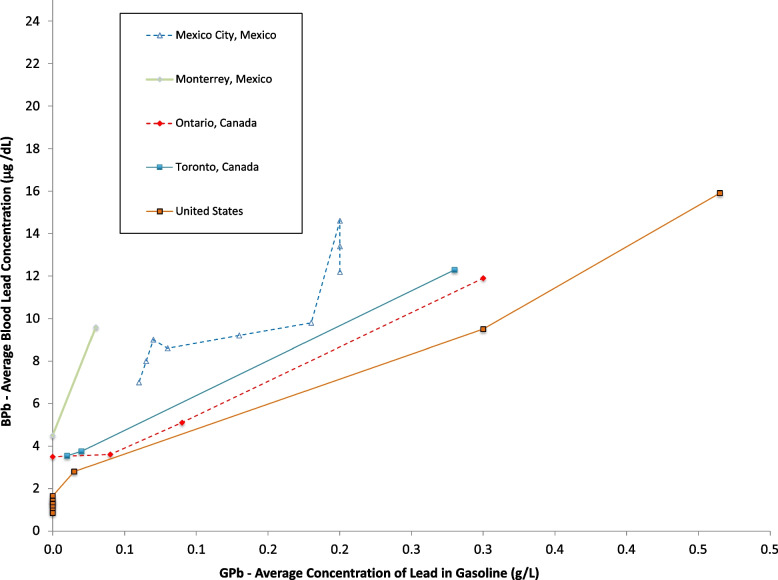
Population blood lead levels versus gasoline lead concentrations in North America. Five studies are shown; each marker is one data point; each line connects the data for a single study

Figures 4 and 5 show data from North America. The data for the United States are the most extensive in North America [66, 67], and show that US blood lead concentrations have fallen below 1 μg/dL as of 2011. Canadian data from Ontario and specifically Toronto are less extensive and somewhat higher than the results from the US overall [68–70]. Studies from Mexico show higher blood lead levels, decreasing over time and decreasing as gasoline lead concentrations decreased [71–74].

**Fig. 6:**
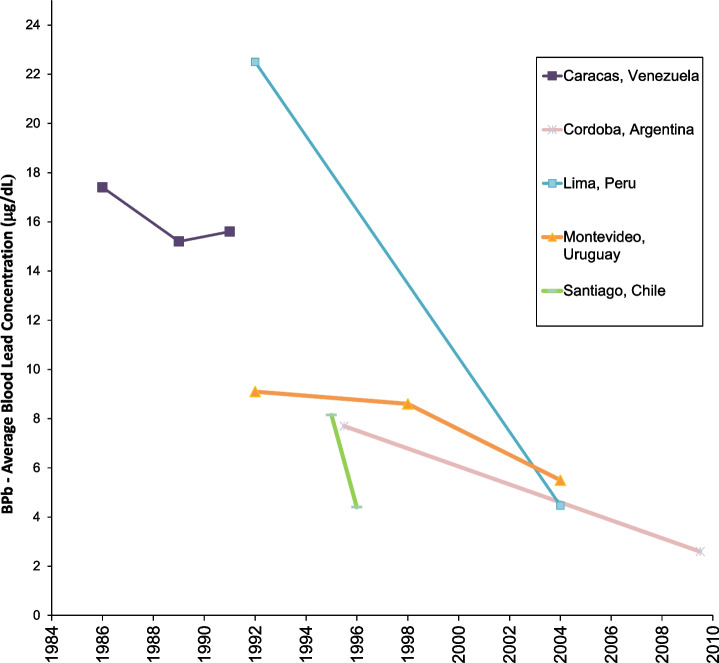
Population blood lead levels by year in South America. Five studies are shown; each marker is one data point; each line connects the data for a single study

**Fig. 7:**
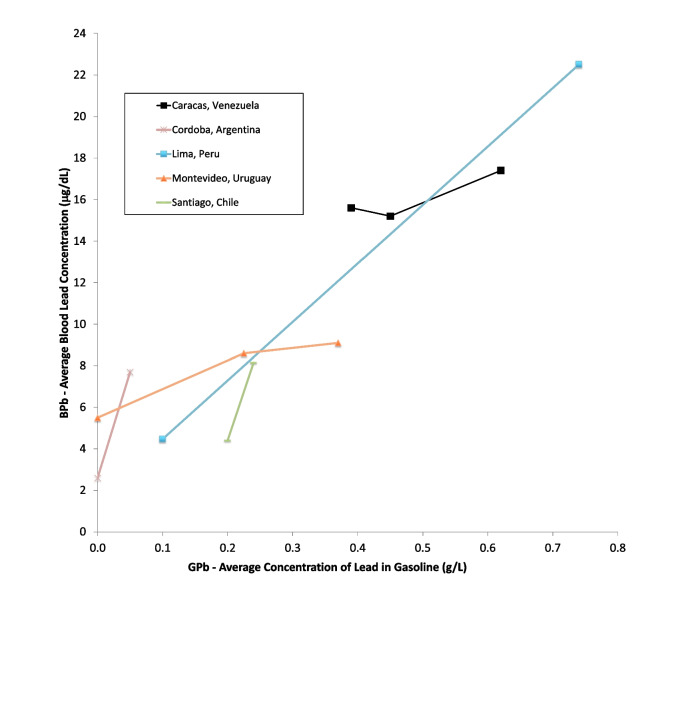
Population blood lead levels versus gasoline lead concentration in South America. Five studies are shown; each marker is one data point; each line connects the data for a single study

Figures 6 and 7 show data from South America. There are no studies in our data set from Central America. The South American studies are from Venezuela, Chile, Uruguay, Argentina, and Peru [75–80]. There are no studies on blood lead concentrations over time from Brazil, Columbia, Ecuador, and others. The lowest population blood lead concentrations reported in these studies is 2.6 μg/dL, in Cordoba, Argentina from 2010. The trajectories shown across the studies suggest that current blood lead levels in Lima, Santiago, and Montevideo may now be lower.

**Fig. 8:**
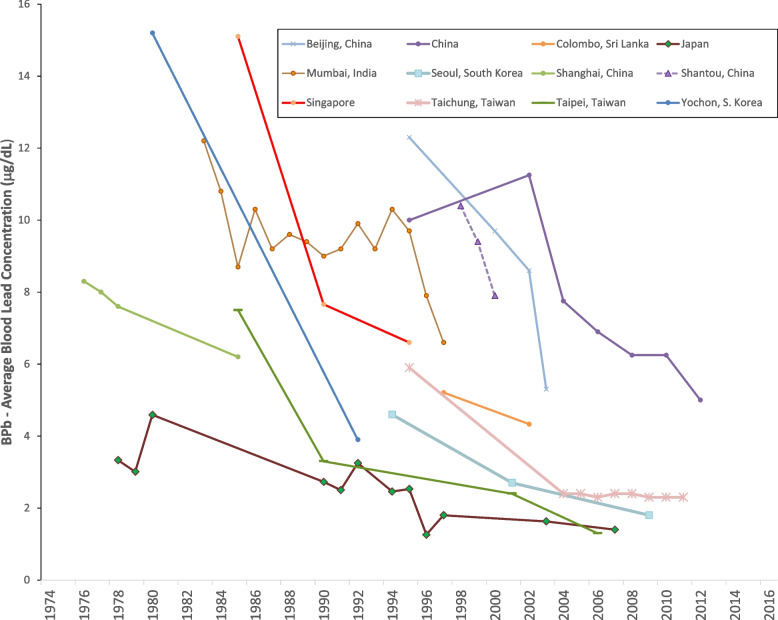
Population blood lead levels by year in Asia. Twelve studies are shown; each marker is one data point; each line connects the data for a single study

**Fig. 9:**
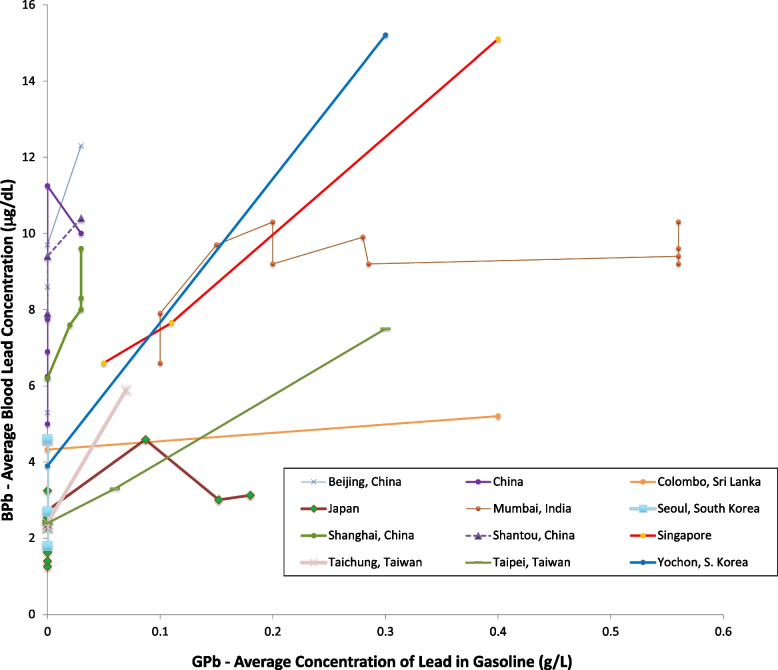
Population blood lead levels versus gasoline lead concentrations in Asia. Twelve studies are shown; each marker is one data point; each line connects the data for a single study

Figures 8 and 9 show the data for Asia. Figure 8 shows that blood lead concentrations have decreased over time in all of the Asian data sets. Figure 9 shows that in most of the countries, decreasing blood lead concentration is associated with decreasing concentrations of lead in gasoline [81–84]. For Mumbai, India, there was substantial decrease in the concentration of lead in gasoline while blood lead levels remained roughly constant. However, between 1985 and 1995, while the concentration of lead in gasoline decreased by approximately a factor of two, the amount of gasoline consumed rose by approximately a factor of [85] two. All of the data sets from China show falling blood lead concentrations with gasoline lead levels already at low levels [86–89]. While data on use of lead in gasoline in China are sparse, there is no indication of heavy use of lead in gasoline. The decreasing blood lead concentrations in China may reflect decreases in other sources of lead exposure. The lowest levels from these studies is 1.4 μg/dL, from Japan in 2008 (Fig. 9) [90–93].

**Fig. 10:**
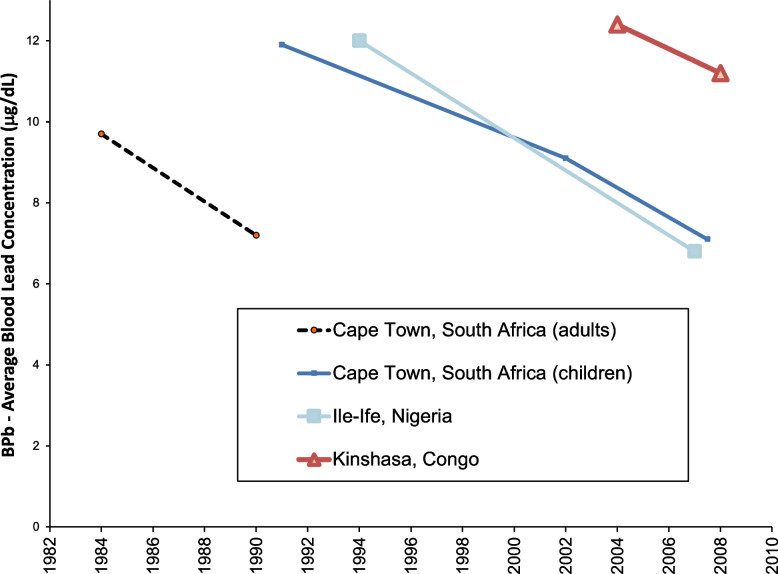
Population blood lead levels by year in Africa. Four studies are shown; each marker is one data point; each line connects the data for a single study

**Fig. 11:**
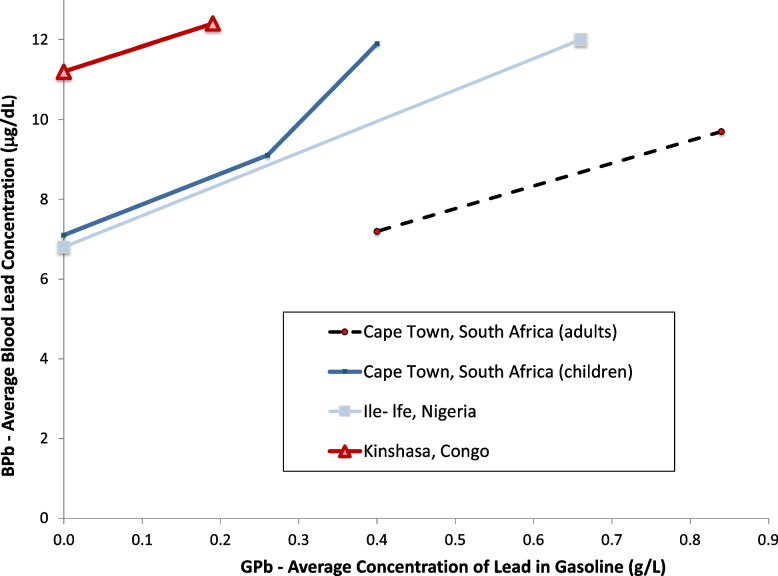
Population blood lead levels versus gasoline lead concentrations in Africa. Four studies are shown; each marker is one data point; each line connects the data for a single study

Figures 10 and 11 show result for Africa, for South Africa, Nigeria, and the Democratic Republic of Congo [94–98]. Although lead was phased out of gasoline in many African countries by 2006, there are ongoing exposures from other sources [31, 99]. The data in Figs. 10 and 11 show consistent slopes, with decreases corresponding to reduced use of lead in gasoline and over time. All of the African studies show relatively lower blood lead concentrations for the gasoline lead concentrations, compared to the results from South America, North American and Europe, while similar to the results from Asia. These may indicate comparatively low consumption of gasoline in the African cities studied, compared to the studies from North and South America and Europe. Further studies of lead exposure in more locations in Africa would be welcome to further examine population lead exposures and trends.

**Fig. 12:**
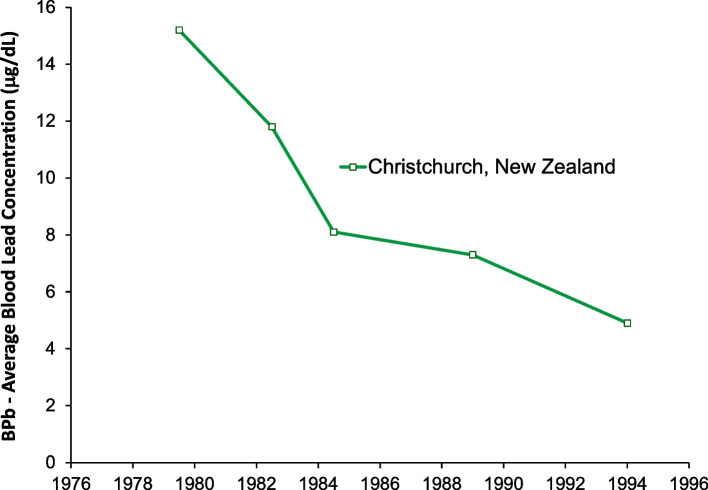
Population blood lead levels versus year in New Zealand. One study is shown; each marker is one data point

**Fig. 13:**
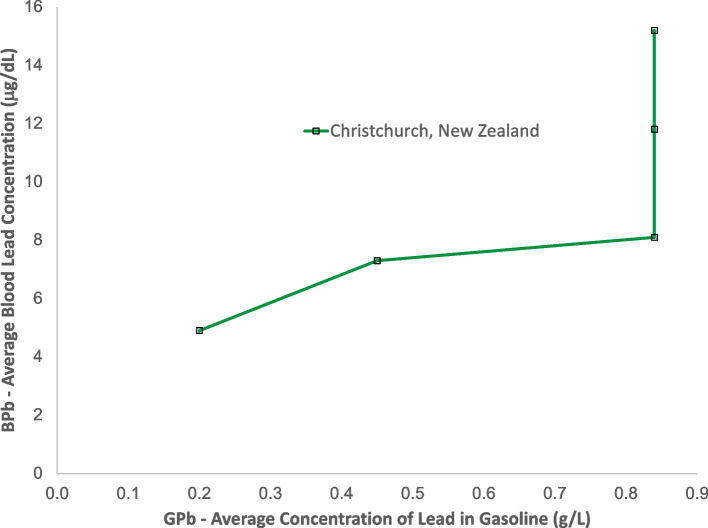
Population blood lead levels versus gasoline lead concentrations in New Zealand. One study is shown; each marker is one data point

Figures 12 and 13 show data from Oceania. This region includes Australia, New Zealand, Melanesia, Micronesia, and Polynesia. There has only been a one published study of blood lead concentration trends in a general population: the study shown from Christchurch New Zealand [100–103]. Australia has been one of the world’s largest producers of lead since the 1800’s, and has the world’s largest lead reserves. Yet there has been no study of population exposure trends. The Christchurch New Zealand study shows a significant decrease in blood lead concentrations from 1978 to 1984, a time during which the gasoline lead concentrations did not change. This may indicate reductions in other sources of lead exposure, or improvements in laboratory measurement techniques. After 1984 the relative values of blood lead versus gasoline lead are similar to those in Europe and in North and South America.

## Discussion

Lead has now been removed from gasoline in all countries in the world. Algeria, the last country to still use lead in motor vehicle gasoline, completed removal in 2021, 99 years after the commercial introduction of tetraethyl lead. These findings corroborate and extend the previous work of Thomas et al., [28] and they confirm that removal of lead from gasoline has been a highly effective strategy for reducing population lead levels. These data show that the reduction in blood lead levels that followed removal of lead from gasoline has been lasting and that the health benefits of lead removal have now been extended to more than two generations of children.

In some cities and countries lead levels have not fallen as sharply as in others. This may speak to widespread population exposure in these locations to lead sources other than gasoline such as lead-based paint, plumbing containing lead (Flint, MI), industrial lead emissions, or lead-containing household and consumer products, for example, widespread use of lead pottery in Mexico, as well as the possibility of systematic measurement errors [29].

There are some cases in which blood lead levels decreased during a time with no known decrease in gasoline lead levels. We see this in the data from Istanbul; Furman [61] reports that there had been a substantial decrease in gasoline lead levels several years before the reported measurements were made. Also, in the United States and several other countries, blood lead levels have continued to fall in the years since the complete phase out of lead in gasoline. These may be due both to decreases in residual exposure to lead from gasoline, and to decreases in other sources of exposure.

The great strength of this study lies in the availability of well curated data sets from multiple cities and countries around the world. The principal weakness is the lack of data from certain areas. There is an ongoing need for measurements of population lead exposure, in low- and middle-income countries [104] as well as in higher income countries. In addition, continuing lead exposures in communities due to legacy or ongoing uses of lead are not reflected in aggregated studies have not received systematic study [105].

## Supplementary Information

**Additional file 1.**

## Data Availability

All data are available in the supplementary information.
