# Supplementary material for: Relation of blood lead levels and lead in gasoline: an updated systematic review
Source: Environ Health. 2022 Dec 27;21:138. doi: 10.1186/s12940-022-00936-x (PMC9793664; doi:10.1186/s12940-022-00936-x)
Supplement: Supplementary file 1 — Additional file 1. [file 12940_2022_936_MOESM1_ESM.zip › 12940_2022_936_MOESM1_ESM.pdf]

Supplemental Material

Figures by continent, and full references are provided in:
Angrand, R.C., Collins, G., Landrigan, P.J., Thomas, V.M. Relation of blood lead levels and lead in gasoline: an updated systematic review. *Environmental Health*. 2022. https://doi.org/10.1186/s12940-022-00936-x

Table S1. Population blood lead concentrations and weighted average gasoline lead concentrations, by year. Alphabetized by city or country name.

Location	Year	Blood Lead (µg/dL)	Gasoline Lead (g/L)	Population Age Range	BPb Reference	GPb Reference
Athens, Greece	1979	—	0.80	adults	Chartsias et al. 1986; Kapaki et al. 1998	
	1982	16.00	0.40			
	1984	11.80	0.22			
	1988	8.00	0.15			
	1993	5.50	0.14			
Barcelona, Spain	1984	18.6	0.60	adults	Rodamilans et al. 1996	
	1994	8.8	0.13			
Beijing, China	1996	12.3	0.03	children	Luo et al. 2012	Octel
	2001	9.7	0.00			
	2003	8.6	0.00			
	2004	5.3	0.00			
Belgium	1979	0.45	17.0	adults	Ducoffre et al. 1990	
	1983	0.40	14.7			
	1987	0.15	9.0			
Bologna, Italy	1980 - 1981	14	0.40	adults	Guberti et al. 1998	Octel
	1984	12.9	0.35			
	1996	5	0.08			
Budapest, Hungary	1986	24.8	0.40	7-9y	Bitto et al. 1997	Bitto et al. 1997
	1992	9.1	0.15	9-10y		
Cape Town, South Africa	1984	9.7	0.84	adults	Maresky and Grobler 1993	Octel
	1990	7.2	0.40			
Cape Town, South Africa	1991	11.9	0.40	7-11y	Matthee et al. 2006	Octel
	2002	9.1	0.26			
	2007 - 2008	7.1			Naicker et al. 2013	
Caracas, Venezuela	1986	17.4	0.62	adults	Cedeno et al. 1990; Romero et al. 1996	
	1989	15.2	0.45			
	1991	15.6	0.39			
China	1996	10	0.03	children	Wang et al. 2015	
	2003	11.25	0.00			

	2005	7.75	0.00			
	2007	6.9	0.00			
	2009	6.25	0.00			
	2011	6.25	0.00			
	2013	5	0.00			
Christchurch, New Zealand	1978 - 1981	0.84	15.2	adults and children	Hinton et al. 1986; Walmsley and Hinton 1998; Walmsley and George 1995; Grant 1996.	
	1982 - 1983	0.84	11.8			
	1984 - 1985	0.84	8.1			
	1989	0.45	7.3			
	1994	0.20	4.9			
Colombo, Sri Lanka	1998	5.21	0.20	1-15y	Senanayake et al. 2004	
	2003	4.33	0.00			
Cordoba, Argentina	1995 - 1996	7.7	0.05	children	Martinez et al. 2013	Martinez et al. 2013
	2009 - 2010	2.6	0.00			
East Germany (Zerbst)	1992 - 1993	3.42	0.03	5-7y	Meyer et al. 2013	Octel
	1995 - 1996	2.65	0.02			
	1998 - 1999	2.38	0.01			
France	1979	13.9	0.40	adults	Huel et al. 2002	Octel
	1982	13.7	0.40			
	1995	7.4	0.08			
Germany	1981	6.15	0.15	adults	Lermen et al, 2021	Octel
	1984	4.5	0.03			
	1985	3.1	0.00			
	1986	6.58	0.15			
	1987	5.55	0.12			
	1988	5.34	0.10			
	1989	5.52	0.08			
	1990	5.47	0.08			
	1991	4.98	0.06			
	1992	3.97	0.02			
	1993	4.39	0.02			
	1995	2.72	0.01			
	1996	2.75	0.00			
	1997	2.11	0.00			
	1998	2.18	0.00			
	1999	2.55	0.00			

	2000	1.87	0.00			
	2001	2.43	0.00			
	2002	1.39	0.00			
	2003	1.72	0.00			
	2004	1.90	0.00			
	2005	1.49	0.00			
	2006	1.52	0.00			
	2007	1.43	0.00			
	2008	1.36	0.00			
	2009	1.29	0.00			
	2010	1.15	0.00			
	2011	1.22	0.00			
	2012	1.24	0.00			
	2013	1.22	0.00			
	2014	1.14	0.00			
	2015	1.20	0.00			
	2016	1.07	0.00			
	2017	1.10	0.00			
	2018	1.13	0.00			
	2019	1.04	0.00			
	2020	0.79	0			
	2021	0.97	0			
Germany	1990-1992	3.2	0.03	6-14y	Kolossa-Gehring et al. 2007	Octel
	2003-2006	1.5	0.00			
Helsinki, Finland	1983	0.35	4.8	children	Pönkä 1993; 1998	
	1988	0.14	3.0			
	1996	0.00	2.6			
Ile-Ife, Nigeria	1994	12	0.66	adults	Ojo et al. 2014	Octel
	2007	6.8	0.00			
Istanbul, Turkey	1993	8.45	0.20	cord blood	Furman and Laleli 2001	Octel
	1998	3.64	0.20			
Japan	1979	3.13	0.18	adults	Zhang et al. 2000; Niisoe et al. 2011; Ikeda et al. 2011	Octel
	1980	3.01	0.152			
	1981	4.59	0.087			
	1991	2.73	0			
	1992	2.5	0			
	1993	3.25	0			
	1995	2.5	0			
	1997	1.79	0			
	1998	1.8	0			
	2004	1.63	0			
	2008	1.4	0			

Landskrona, Sweden	1978	6.0	0.15	children	Strömberg et al. 2008	
	1982	4.8	0.15			
	1984	3.9	0.15			
	1986	4.1	0.15			
	1988	3.3				
	1990	3.6				
	1991	3.3				
	1992	3.0	0.06			
	1994	2.5	0.01			
	1996	2.3	0.00			
	1998	2.3	0.00			
	2000	2.2	0.00			
	2002	1.9	0.00			
	2004	1.8	0.00			
	2006	1.4	0.00			
	2007	1.32	0.00			
Kansai (Kyoto), Japan	1980	16.2	0.15	newborns	Sugiyama et al. 1996	Octel
	1990	4.9	0.00			
Kinshasa, Congo	2004	12.4	0.19	0-5y	Tuakuila et al. 2013	Octel
	2008	11.2	0.00			
La Spezia, Italy	1990	14	0.15	traffic wardens	Neri and Palmieri 1998	Octel
	1993	8.5	0.11			
	1996	7.2	0.08			
Lima, Peru	1992	22.5	0.74	profession al drivers	Mormontoy et al. 2006	Octel
	2004	4.47	0.10			
Madrid, Spain	1995	3.8	0.11	0-15y	Ordoniz-Iriarte et al. 2015	
	2010	0.9	0.00			
Mexico City, Mexico	1988	0.20	12.2	children	Rothenberg et al. 1998; Driscoll et al. 1992; Mexico City, 1993.	
	1989	0.20	14.6			
	1990	0.18	9.8			
	1991	0.08	8.6			
	1992	0.07	9.0			
	1993	0.06	7.0			
Monterrey, Mexico	1998	9.6	0.03	6-12y	Santos-Goodman et al. 2014	
	2008	4.5	0.00			
Montevideo, Uruguay	1992	9.1	0.37	adults	Cousillas et al. 2012	Octel
	1998	8.6	0.225			
	2004	5.5	0			
Mumbai, India	1984	12.2	0.29	6-10y	Tripathi et al. 2001	Octel
	1990	9.4	0.36			
	1991	9	0.36			

	1992	9.2	0.29			
	1993	9.9	0.29			
	1994	9.2	0.22			
	1995	10.3	0.22			
	1998	6.6	0.01			
Ontario, Canada	1984	0.30	11.9	children	Langlois et al. 1996; Loranger and Zayed 1994	
	1988	0.09	5.1			
	1990	0.04	3.6			
	1992	0.00	3.5			
Porto, Portugal	1992	10.4	0.30	newborns	Roma-Torres et al. 2007	Octel
	2001	2.2	0.00			
Santiago, Chile	1995	8.15	0.24	1y	Pino et al. 2004	Octel
	1996	4.4	0.20			
Seoul, S. Korea	1995	4.6	0		Niisoe et al. 2011	Octel
	2002	2.7	0			
	2010	1.8	0			
Shanghai, China	1995	9.6	0.03	children	Luo et al. 2012	Octel
	1997	8.3	0.03			
	1998	8	0.03			
	1999	7.6	0.02			
	2006	6.2	0.00			
Shantou, China	1999	10.4	0.03	1-5y	Luo et al. 2003	Literature
	2000	9.4	0.00			
	2001	7.9	0.00			
Silesia, Poland	1993	6.8	0.14	3-5y	Jarosinska et al. 2006	Octel
	1994	6.9	0.12			
	1995	6.1	0.09			
	1996	6.8	0.09			
	1997	5.2	0.07			
Singapore	1986	15.1	0.40	adults	Neo et al. 2000	Octel
	1990 -1	7.66	0.11			
	1996	6.6	0.05			
Southern Sweden	1978	6.9	0.36	7-12 y	Skerfving 2015	
	1980	5.2	0.15			
	1982	5.2	0.15			
	1984	4.5	0.06			
	1986	4.5	0.01			
	1988	3.5	0.008			
	1990	3.8	0			
	1992	3.2	0			
	1994	2.9	0			
	1996	2.4	0			

	1998	2.5	0			
	2000	2.5	0			
	2002	2.2	0			
	2004	1.9	0			
	2006	1.5	0			
Stockholm, Sweden	1980	0.40	7.7	adults	Elinder et al. 1986	
	1983	0.15	5.4			
Switzerland	1984 - 1985	0.15	10.3	adults	Wietlisbach et al. 1995	
	1988 - 1989	0.10	7.3			
	1992 - 1993	0.05	5.9			
Taichung, Taiwan	1994 -5	0.07	5.9			
	2005	0	2.4			
	2006	0	2.4			
	2007	0	2.3			
	2008	0	2.4			
	2009	0	2.4			
	2010	0	2.3			
	2011	0	2.3			
	2012	0	2.3			
Taipei, Taiwan	1985 - 1987	0.30	7.5	cord blood	Hwang et al. 2004	
	1990 - 1992	0.06	3.3			
	2001 - 2002	0.00	2.4			
Tarragona, Spain	1990	0.40	12.0	adults and children	Schumacher et al. 1996	
	1995	0.13	6.3			
Toronto, Canada	1984	12.3	0.28	1-6y	Wang et al. 1997	Octel
	1990	3.7	0.02			
	1992	3.5	0.01			
Trelleborg, Sweden	1979	5.6	0.15	children	Strömberg et al. 2008	
	1980	4.8	0.15			
	1981	5.25	0.15			
	1983	4.1	0.15			
	1991	2.75	0.06			
	1993	2.3	0.01			
	1995	2.2	0.00			
	1997	1.85	0.00			
	1999	2.15	0.00			
	2001	1.8	0.00			

	2003	1.6	0			
	2005	1.3	0			
Turin, Italy	1980	0.60	21.0	adults	Bono et al. 1995	
	1985	0.40	15.1			
	1989	0.30	—			
	1993	0.11	6.4			
Umbria, Italy	1982	19.65	0.35	adults	Picccinini et al. 1998	Octel
	1992	7.95	0.13			
United Kingdom	1979	0.42	12.9	adults and children	Quinn 1985; UK DOE 1987; Delves et al. 1996	
	1981	0.38	11.4			
	1985	0.38	9.5			
	1986	0.14	8.4			
	1995	0.055	3.1			
United States	1976	15.9	0.465	population	Annest et al. 1983; Pirkle et al. 1985; Tsoi et al. 2016, Tsoi et al. 2016; CDC 2018	
	1977	14.0	0.394			
	1978	14.6	0.349			
	1979	12.1	0.306			
	1980	9.5	0.30			
	1988 - 1991	2.8	0.00			
	1999-2000	1.65	0.00			
	2001-2002	1.44	0.00			
	2003-2004	1.43	0.00			
	2005-2006	1.29	0.00			
	2007-2008	1.27	0.00			
	2009-2010	1.12	0.00			
	2011-2012	0.97	0.00			
	2013-2014	0.86	0.00			
	2015-2016	0.82	0.00			
	2017-2018	0.753	0.00			
Venice, Italy	1976	25.8	0.48	adults and children	Bortoli et al. 1998	Octel
	1979	16.67	0.47			
	1981	15.05	0.37			
	1985 -6	10.43	0.35			
	1992 -3	5.47	0.12			
Yochon, S. Korea	1981	15.2	0.30		Kim et al. 2011	Octel
	1993	3.9	0.00			
Zagreb, Croatia	1981	13.7	0.60	adult males	Zorana et al. 2016	

	1989	10.5	0.60			
	1998	6	0.50			
	1999	5.8	0.50			
	2001	5.5	0.50			
	2003	3.6	0.15			
	2004	2.2	0.15			
	2005	3.6	0.15			
	2006	3.3	0.00			
	2007	3	0.00			
	2008	2.9	0.00			
	2009	2.8	0.00			
