# Supplementary figures and images for: Relation of blood lead levels and lead in gasoline: an updated systematic review
Source: Environ Health. 2022 Dec 27;21:138. doi: 10.1186/s12940-022-00936-x (PMC9793664; doi:10.1186/s12940-022-00936-x)

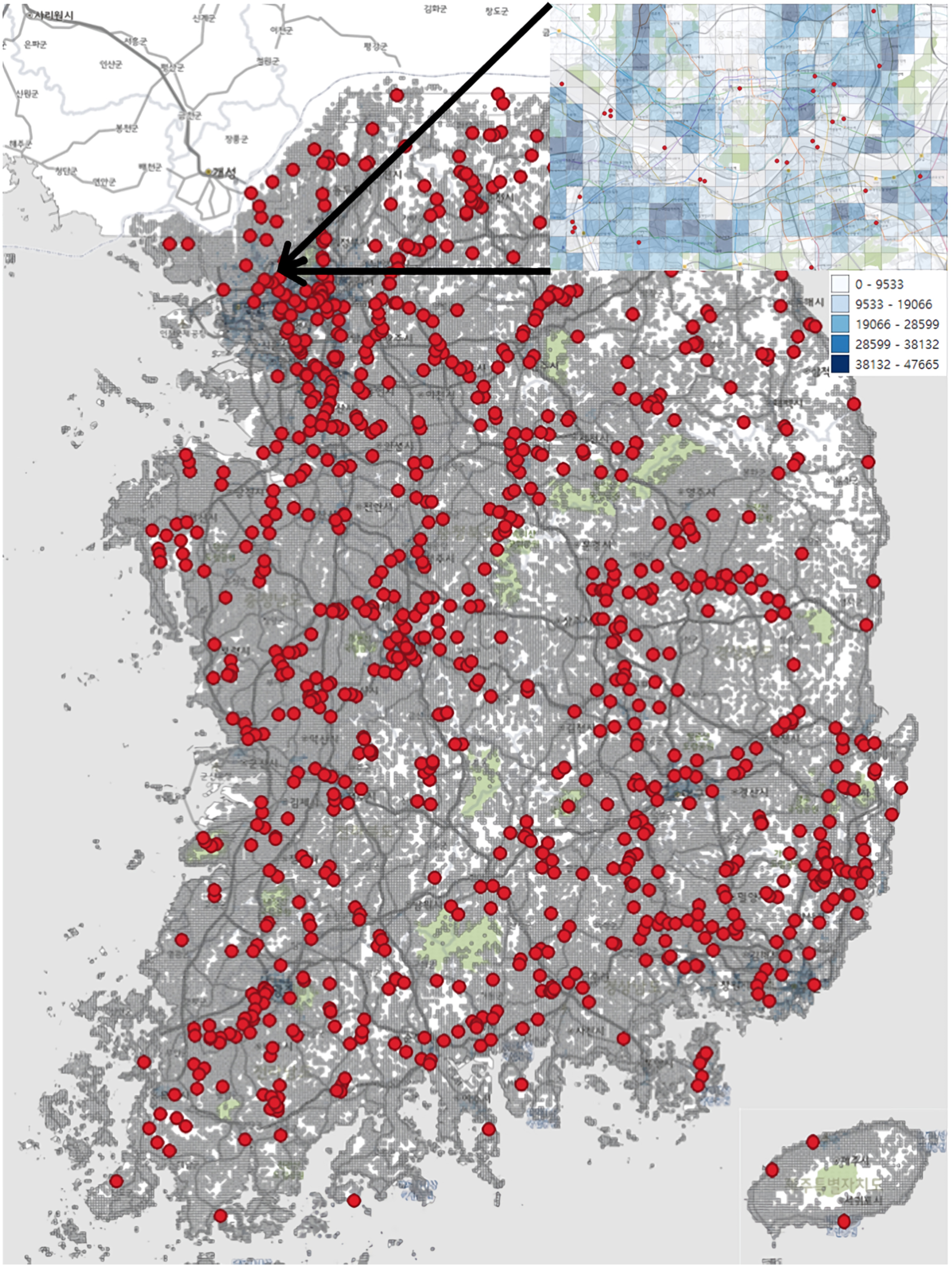

Supplement: Supplementary file 1 — Additional file 1. [file 12940_2022_936_MOESM1_ESM.zip › 12940_2022_936_Fig1.tif]
